# The Prevalence and Trends of Hepatitis B, Hepatitis C, and HIV among Voluntary Blood Donors in Kohgiluyeh and Boyer-Ahmad Transfusion Center, Southwestern Iran

**Published:** 2018-07

**Authors:** Seyed Mehdi SAJJADI, Ali Akbar POURFATHOLLAH, Saeed MOHAMMADI, Bijan NOURI, Rostam HASSANZADEH, Fariba RAD

**Affiliations:** 1. Cellular and Molecular Research Center, Birjand University of Medical Sciences, Birjand, Iran; 2. Iranian Blood Transfusion Research Center, High Institute for Research and Education in Transfusion Medicine, Tehran, Iran; 3. Hematology, Oncology and Stem Cell Transplantation Research Center, Tehran University of Medical Sciences, Tehran, Iran; 4. Social Determinants of Health Research Center, Kurdistan University of Medical Sciences, Sanandaj, Iran; 5. Cellular and Molecular Research Center, Yasuj University of Medical Sciences, Yasuj, Iran

**Keywords:** Transfusion transmissible infections, Prevalence, Trend, Iran

## Abstract

**Background:**

Transfusion transmissible infections (TTIs) are a common complication of blood transfusion. Evaluation and monitoring the prevalence rate of TTIs in blood donors is a valuable indicator of donor selection and blood safety. We analyzed the trends of these infections among blood donors at Kohgiluyeh and Boyer-Ahmad transfusion service (KBTC) during 10 years.

**Methods:**

Viral screening and confirmatory tests were carried out on 180304 voluntary donations from 2005–2014. The annual prevalence rates of hepatitis B virus (HBV), hepatitis C virus (HCV) and HIV infections per 100000 donations and 95% confidence interval were calculated. Chi-square test was applied to obtain the *P*-value.

**Results:**

The overall prevalence was 0.13% for HBV and 0.06% for HCV while there were only three positive cases for HIV. The annual trend fluctuated during the time period studied. Compared to first-time donors, regular and repeat donors were significantly less likely to be positive for these infections. Outstandingly, this study provides first data in TTIs seropositivity rates among blood donors in our region; surprisingly were lower compared to other reports of Iran.

**Conclusion:**

The trends of TTIs prevalence in this study provide additional evidence that safety measures employed by the KBTC have been effective in maintaining a safe blood supply. The lower prevalence of TTIs in our study compared with other Iranian studies and also the general population reflects the efficacy of donor selection and education procedures in KBTC.

## Introduction

Each year, more than 90 million blood units are collected in blood transfusion centers around the world (1); one-third of the world population will require blood transfusions or blood products at some point during their lifetimes. Blood transfusions do save millions of lives but unhealthy blood transfusions can have catastrophic health consequences on recipients, their families and on society in terms of economic costs (1, 2). Thus, providing safe blood and blood products is a universal concern (3). Any recipient is at risk of transfusion-transmissible infections (TTIs), including the human immunodeficiency virus (HIV), hepatitis B (HBV) and hepatitis C (HCV) (1). Among these, HBV and HCV infections represent a special concern (4) due to the higher risk of transmission via blood and blood products (5). HBV is one of the most common sources of chronic liver morbidity (6) while HCV is the major cause of post-transfusion hepatitis which, can result in chronic infections, cirrhosis and hepatocellular carcinoma (7).

The worldwide prevalence of HBV and HCV in carrier status is estimated at about 350 million and 150 million infections, respectively (4). “In Iran, screening of blood donations for the HBV surface antigen (HBsAg), HIV and HCV became mandatory in 1974, 1989 and 1996, respectively” (8). Although, the rate of TTI transmission in recipients has been reduced drastically by employing the pre-donation questionnaires, donor education, voluntary donation, donor deferral and the use of advanced donor screening assays (4, 9–11), it has not been completely eliminated due to factors such as genetic variations in infectious agents, silent carriers, laboratory errors and variations in window periods (4, 12, 13). Therefore, these infections remain an important public health problem; special attention is being given to ensuring that blood transfusion organizations, particularly in developing countries, are making plans to decrease the prevalence trends of these infections (2). Evaluating TTI trend is a critical approach to monitoring the effectiveness of donor education, selection, and screening methods, leading both to supplying safe blood resources and obtaining a good estimate on the epidemiology of these infections in the population (3, 7, 14).

The aim of this 10-year retrospective study was to assess the changes in prevalence trends of TTI infections among first-time, regular and repeat blood donors who attended the Kohgiluyeh and Boyer-Ahmad transfusion service, Kohgiluyeh and Boyer-Ahmad Province, Iran, from 2005–2014. To the best of our knowledge, no previous study of TTI prevalence trends over time had been carried out in the current study population.

## Materials and Methods

This retrospective study was conducted at the Kohgiluyeh and Boyer-Ahmad Blood Transfusion Center (KBTC). All voluntary, non-remunerated blood donors who donated blood from 2005–2014 were investigated. The donors were selected based on the standard pre-donation screening process, including a health history questionnaire and physical examination. Donors were divided into three groups: first-time donors who were donating for the first and only time; regular donors who donated more than once during a year; and repeat donors who had a history of the previous donation, but the interval between two donations was longer than a year.

All 180304 donated units were screened for HBsAg, HCV Ab and HIV (Ag/Ab) by ELISA based on IBTO instructions. HBsAg was detected using Behring, Simens, and Biorad kits, anti-HCV was detected by Orto, Biomerieux, Hepanostika, Biomedical, and Murex kits and HIV-Ab was detected by Vironostika, Biomerieux, Biorad, and Adaltis kits. The initially reactive samples were evaluated again; the repeatedly reactive ones were considered seropositive. Hepatitis B core antibody (anti-HBc) and HBsAg neutralization tests (Behring, Marburg, Germany), HCV recombinant immunoblot assay (RIBA) (Inonogenetic, Ghent, Belgium) and HIV western blots (Inonogenetic, Ghent, Belgium) were undertaken for all repeatedly positive samples. HIV p24 antigen was done for the HIV western blot -negative samples and monoclonal neutralization assay was carried out if the results were repeatedly reactive.

Statistical analysis was performed using SPSS software (ver. 16.0, Chicago, IL, USA). To evaluate the infection trends, the prevalence of TTIs per 100000 donations was reported separately among first-time, regular and repeat blood donors.

## Results

Over a period of 10 yr (2005 to 2014), a total of 180304 donations were collected from volunteer donors, with a mean of 18030.4 donations per year. 10418 donors (5.77%) were females and 169886 (94.23%) were males, leading to a male-to-female ratio of 16.3 to 1. Screening tests were done for all donations according to the Iranian Blood Transfusion Organisation (IBTO) guidelines. The annual prevalence rates (PRs) per 100000 for these infections from 2005–2014 are presented in Tables 1, 2.

**Table 1:** The annual prevalence rates (PRs) per 100,000 donations for HBV infection among first, regular, and repeat blood donors at Kohgiluyeh and Boyer-Ahmad transfusion service from 2005 to 2014

***Year***	***HBV***											
	***No of Donations***				***No***				***PR/10^5^***			
	***Total***	***First***	***Regular***	***Repeat***	***Total***	***First***	***Regular***	***Repeat***	***Total***	***First***	***Regular***	***Repeat***
2014	23743	4238	5489	14016	39	38	0	1	34	897	0	7
2013	23880	5121	4888	13871	20	18	2	0	21	351	41	0
2012	25540	7069	4649	13822	21	20	1	0	43	283	22	0
2011	22587	7482	3601	11504	23	22	1	0	44	294	28	0
2010	17094	6108	3223	7763	15	14	1	0	29	229	31	0
2009	13845	3320	2881	7644	23	19	2	2	130	572	69	26
2008	13670	3127	2534	8009	34	31	0	3	176	991	0	37
2007	13712	3505	2140	8067	35	33	0	2	88	942	0	25
2006	13270	4143	1544	7583	21	20	1	0	83	483	65	0
2005	12963	5720	762	6481	19	18	0	1	85	315	0	15

**Table 2:** The annual prevalence rates (PRs) per 100,000 donations for HCV infection among first, regular, and repeat blood donors at Kohgiluyeh and Boyer-Ahmad transfusion service from 2005 to 2014.

***Year***	***HCV***											
	***No of Donations***				***No***				***PR/10^5^***			
	***Total***	***First***	***Regular***	***Repeat***	***Total***	***First***	***Regular***	***Repeat***	***Total***	***First***	***Regular***	***Repeat***
2014	23743	4238	5489	14016	8	8	0	0	34	189	0	0
2013	23880	5121	4888	13871	5	4	0	1	21	78	0	7
2012	25540	7069	4649	13822	11	7	3	1	43	99	65	7
2011	22587	7482	3601	11504	10	9	1	0	44	120	28	0
2010	17094	6108	3223	7763	5	3	2	0	29	49	62	0
2009	13845	3320	2881	7644	18	17	1	0	130	512	35	0
2008	13670	3127	2534	8009	24	22	1	1	176	704	39	12
2007	13712	3505	2140	8067	12	11	0	1	88	314	0	12
2006	13270	4143	1544	7583	11	9	1	1	83	217	65	13
2005	12963	5720	762	6481	11	10	1	0	85	175	131	0

Among all donors, 250 were found to be positive for HBsAg (0.13%) and 115 for HCV (0.06%), while there were only three, confirmed positive results for HIV. The prevalence of HBV infection among first-time donors increased from 315 per 100000 donations in 2005 to 991 per 100000 donations in 2008 (RR, 0.95; 95% CI, 0.93–0.99; *P*=0.011) (one-sample *t*-test, *P*<0.001), which was statistically significant before dropping to 229 per 100000 in 2010. From 2010 to 2013, no clear annual trend was observed in first-time donors; however, an increase was seen again from 351 per 100000 donations in 2013 to 897 per 100000 donations in 2014 (RR, 0.95; 95% CI, 0.93–0.99; *P*=0.011). Compared to first-time donors, regular and repeat donors were significantly less likely to be positive for the HBV infection (RR, 0.03; 95% CI, 0.02–0.05; *P*<0.001).

A similar pattern was observed in HCV infection, in which the numbers increased from 217 per 100000 donations in 2005 to 704 per 100000 donations in 2008 but decreased after that to 229 per 100000 donations in 2010. As with HBV infection, no clear annual trend was observed in first-time donors from 2010 to 2013, but the HCV PR increased to 189 per 100000 donations in 2014 (RR, 0.95; 95% CI, 0.93–0.99; *P*=0.011).

From 2005 to 2014, HCV prevalence in regular donors varied between 131 and 0.0 per 100000 donations, with no clear annual trend (RR, 0.99; 95% CI, 0.95–1.04; *P*=0.65). As was expected, the lowest PR for HCV was obtained for repeat donors, with the maximum number of seropositive donations for HCV Ab being 13 per 100000 donations in 2006–2007. The majority of HBV- and HCV-positive donors from 2005 to 2014 were first-time rather than regular and repeat donors (93.2% and 86.95%, respectively). The most frequent infection in first-time donors was HBV (69.34%), followed by HCV (29.76%), which together represented more than 99% of all positive first-time donations. Since there were only three cases of HIV, all from first-time donors in 2012, no trends were observed for this infection.

## Discussion

In this study, the overall frequency of HBV and HCV infections in volunteer blood donors referred to the KBTC was 0.13% and 0.06% respectively. In our study, HBV prevalence among first-time blood donors showed a significant upward trend from 2005–2008. In the following years, the prevalence declined and then remained relatively stable from 2010–2013. However, it increased significantly again in 2014.

Our result was inconsistent with previously reported trends in Iran. In this regard, a downward trend was indicated for HBV from 1998 through 2007, and from 2004 through 2007. The prevalence of HBV in the country as a whole and in a very low-prevalence region, Fars, was 0.4% and 0.34% in 2007 respectively (2). The total prevalence of HBV was also reported as 558 per 100000 donations (8). Similar declines in HBV infection among blood donors have been reported from 2003–2005 and another study from 2005–2011; they reported, respectively, 600 per 100000 and 338 per 100000 confirmed HBs-Ag cases respectively in Tehran’s blood transfusion center (3, 15). There are other studies with similar results from the United Kingdom (1993–2001) (16), the United States (1995–2002) (17) and Turkey (1989–2004) (18). On the contrary, a distinct increase of 1.28% to 1.66% (2004–2005) has been reported in Eastern India (19). Compared to all of these reports, different rates were detected in our study. We found an increasing trend from 2005–2008, which then dropped from 2008–2010. Moreover, in our study, HBV prevalence in 2007 was 0.25%, while overall prevalence during the decade studied was 139 per 100000 donations, of which are both lower. HBV infection distributions show discrepancies across national borderland even between different regions in a country (11). This divergence could be due to variable numbers of donors, different years under study, different local epidemiological situations and the people constituting the study population, who move to different areas from time to time.

However, we think that the increase in HBs Ag positive during 2005 to 2008 may be related to the system of acceptance of blood donors. Maybe they did not attend to valid questionnaire in asking the first blood donors suitably and maybe they accepted more HBs Ag positive in their blood donors and this increase repeated again 2013–2014. We think it was a problem in data or method of interview with volunteer donors.

Our result is lower than the mean prevalence of 1.7% (20) and 2.8% (21) in the general Iranian population because donors are a self-selected group at lower risk of infectious diseases. In comparison with reports in other countries, HBV prevalence in our population was much lower than what has been found in studies performed in India (0.66%) (22), the Pakistani Punjab (4.93%) (23), Turkey (2.1%) (18), Nigeria (7.50%) (24), Yemen (9.8%) (25) and Ethiopia (25%) (26). However, our reported figure was higher than Australia, which had a rate of only 0.01% (27). As in our findings, HBV was the most common infection identified among first-time donors in the United Kingdom and Australia (7, 28). Overall, the findings of this study provide additional evidence that the procedures employed by the KBTC are very effective in maintaining a safe blood supply. Given the increasing trends of HBV and HCV among first-time donors in 2014, data on demographic and risk factors should be collected from positive cases (7).

With less than 1% HCV prevalence in the general population, Iran is considered a country with low incidence of the infection (29). HCV prevalence varies noticeably in different regions of Iran (30). In our study, the overall frequency of HCV during the ten years from 2005 to 2014 was determined to be 0.06%. That was lower than in other studies conducted on Iranian blood donors (3, 7, 8). The pooled prevalence rate of HCV among Iranian blood donors in different provinces and cities was 0.5%. However, variable results were obtained (32). The finding of the present study is inconsistent with other countries, such as Ethiopia with 13.3% (27), Pakistan with 4.06% (23), Cameron with 4.8% (33), Nigeria with 0.86% (24) and India with 0.84% (19).

Furthermore, our finding was lower than the prevalence of HCV infection in the general population in Iran, as expected (21). Similar to HBV, an increasing trend of HCV prevalence was observed among first-time blood donors during 2005–2008, and then it declined significantly during 2008–2010. During the subsequent time period studied, it fluctuated, peaking in 2014. This increasing pattern in 2014, also seen for HBV, might be due to several issues, including the use of different screening reagents, changes in population risk factors and improvements in the donor screening procedure. In addition, physicians’ incentives were reduced due to various reasons, such as insufficient salary so, as they did not take enough time for the patients’ examinations, which could result in the TTI increment. Now, IBTO has chosen some strategies to increase their incentives. Increased cultural diversity and the expansion of relations with other societies, especially Persian Gulf countries, may be other reasons for the TTI increase in our population. These factors may have led to an increase in the number of confirmed positives. Furthermore, the low number of blood donations may also have biased the results. In this study, the trend in prevalence’s of TTIs was in accordance with the confidential unit exclusion rate. In other words, with reduction in CFU, the prevalence’s were reduced while the increase in CFU resulted in higher prevalences.

A gradual decline in HCV prevalence in blood donations from, 0.14% in 2005 to 0.12% in 2007 was found (8). In comparison with the results obtained in other countries, the declining trend in HCV prevalence was ascribed among Canadian donors during 1993–2006 to a distinct reduction of HCV infection among first-time donors (34). A similar declining trend was reported among blood donors in the United States from 1999–2008 (35).

Moreover, our study showed that HBs-Ag and HCV positivity rates among regular and repeat donors were significantly lower than for first-time donors. This is consistent with other studies (8, 36). However, the observed difference in prevalence rates is not unexpected, due to the removal of positive cases among regular and repeat donors by donor selection criteria or testing. Similar findings that first-time donors show higher rates of infections were also reported in other studies in Australia (33), the United Kingdom (37) and the Netherlands (38). Voluntary, regular and repeat blood donors constitute the most trustworthy group for transfusion safety (38, 39). The number of repeat blood donors was much higher than the number of first-time blood donors (7, 40). However, we obtained different results: more than 30% of our donations were from first-time donors (41).

In this study, only three of the 180304 subjects tested for HIV were found to be positive. This equates to a rate of 1.66 per 100000 donations for first-time blood donors in 2012. In other Iranian studies, the prevalence rates of HIV infection were about 4.0, 4.4 and 5.4 per 100000 donations, respectively. The prevalence of HIV infection in the Iranian general population was reported as about 8.6 per 100000 in 1999 and 23 per 100000 in 2007, both higher than our result (41). Compared to European countries with a prevalence of about 10 per 100000 donations, our finding was very low (42). Furthermore, it was lower than that of other countries such as Nigeria with 0.96% (24), Turkey with 0.20% (43) and India with 0.39% (22); however, a negative result was indicated for HIV in Punjab, Pakistan (23).

The considerable differences in the prevalence of these infections among blood donors in different studies might be due to differences in the study population, sample sizes, study periods, the geographic regions, risk factors, methods used, the assay reagents and kits employed to determine the infectious agent (30). Moreover, we think that this lower prevalence of HIV in our study may be due to the traditional culture in Kohgiluyeh and Boyer-Ahmad Province so that, people believe sexual morality and avoiding of high-risk behaviors such as sexual relations with different partners before marriage.

## Conclusion

A number of factors may have also attributed to declining prevalence: immunization against HBV; improved recruitment of low risk donors; the setup of confidential unit exclusion (CFU); the usage of a computerized data registry of blood donors for transfusion services; an increase in public knowledge of transfusion-transmissible infections, risk factors and transmission routes; improving public health programs; an increase in voluntary blood donation to 100%; an increasing number of regular/repeat donations; improvements in automation and using highly sensitive screening test kits. Despite all these endeavors, it is not feasible to eradicate the risks of TTIs.

## Ethical considerations

Ethical issues (Including plagiarism, informed consent, misconduct, data fabrication and/or falsification, double publication and/or submission, redundancy, etc.) have been completely observed by the authors.
